# Patience

**Published:** 1919-12-27

**Authors:** 


### PATIENCE.

"*Ierne Lacks Patience.*"—The Hospital.

The man of patience is very often the man whose motto is "to-morrow":

"And all our yesterdays have lighted fools
The way to dusty death."

Personally, I admit the soft impeachment that I lack patience. I cannot help it. I am so built up. I may not be here to-morrow, and, if I am, there is still staring me in the face to-morrow, and

"To-morrow's falser than the former day."

A true nurse, we are told, is a woman with a vocation. She was born to nurse, and nurse she must. She cannot continually nurse the sick, and so in her spare time she must nurse her patience. I have recently come across not a few who, having nursed the sick for full twenty years, and their patience in their off time, are now nursing their wrath. It is beginning to dawn on them that a good time, although it is really and truly coming, is not likely to arrive in their day. "What has posterity done for us?" they ask,

"That we, lest they their rights should lose,
Should trust our necks to grip of noose?"

I put it to you, reader, what is the proper and orthodox gospel for the nurse—is it to-day, or to-morrow? Above all things, I like to be specific, and I like everybody else to be specific also, and it seems to me that the gospel must be either to-day or to-morrow. I cherish a certain secret admiration for the tactics of the importunate widow. I presume it was a widow! She was in dire need, and the old gentleman who had it in his power to minister to her necessity was asleep. We all know, from sore experience, how iniquitous a thing it is to hear continual hammering when the ravelled sleeve of care is being knitted up. I reckon that the old man ejaculated some before he passed out the loaf.

Now, my conception of the position may be erroneous, but it is specific, and it is this: First and foremost, the British nurse's hour is to-day. She has spent her forty years in the wilderness, and her right is the Promised Land, not to-morrow, but here and now. I can conceive of no more opportune moment for achievement. And, although it may not be apparent, I am equally concerned for the hospital governors. They do not want to descend into a lower grade for candidate probationers. They want women of refinement and education. They want the nation's best. I am as convinced that they cannot hope to find what they want by preaching any gospel of patience to women who have worn out their lives in devotion to the nation.

The nation must be awakened. IERNE.

